# Case Report of Nasal Rhinosporidiosis in South Africa

**DOI:** 10.3201/eid3004.240018

**Published:** 2024-04

**Authors:** Huzaifah Mayet, Denasha L. Reddy, Tika Bello Alvarez, Yahya Atiya, Nelesh P. Govender, Monica Birkhead, Tsidiso Maphanga, Sugeshnee Pather

**Affiliations:** University of the Witwatersrand, Johannesburg, South Africa (H. Mayet, D.L. Reddy, T. Bello Alvarez, Y. Atiya, N.P. Govender, S. Pather);; Chris Hani Baragwanath Academic Hospital, Johannesburg (H. Mayet, D.L. Reddy, T. Bello Alvarez, Y. Atiya, S. Pather);; St George’s University of London, London, UK (N.P. Govender);; University of Cape Town, Cape Town, South Africa (N.P. Govender);; University of Exeter, Exeter, UK (N.P. Govender);; National Institute for Communicable Diseases, Johannesburg (N.P. Govender, M. Birkhead, T. Maphanga);; National Health Laboratory Service, Johannesburg (S. Pather)

**Keywords:** rhinosporidiosis, Rhinosporidium, fungi, eukaryotic pathogens, aquatic protistan parasites, nasal polyps, histology, PCR, scanning electron microscopy, South Africa

## Abstract

We describe a classic case of nasal rhinosporidiosis in a woman who resided in Johannesburg, South Africa, but originated from a rural area in Eastern Cape Province. We confirmed histologic diagnosis using PCR testing and compared details with those from records on 17 other cases from South Africa.

A 24-year-old Black woman from South Africa sought care at a local primary-level clinic in Soweto, Johannesburg, South Africa, reporting a painless nasal mass of 3 years duration that caused occasional difficulty in breathing. The patient resided in Soweto but was originally from Mqunduli (31°49′S, 28°45′E), a riverside village south of Mthatha, Eastern Cape Province, South Africa. The patient reported the mass had originated in her right nostril; she disclaimed any preceding trauma and described recent onset of pain and intermittent episodes of mild, self-limiting bleeding on contact (e.g., an accidental bump) at the site of the mass. She had no rhinorrhea, and her vision was normal. 

The woman was treated for sinusitis for 1 month but 2 months after initially seeking treatment was referred to the otorhinolaryngology clinic at a tertiary academic facility, where we examined her. We report details of her condition, diagnosis, treatment, and outcomes. We obtained written informed consent from the patient for publication of an account of her case including use of clinical photographs and ethics clearance from the University of the Witwatersrand Human Research Ethics Committee (M210752). 

## The Study

We diagnosed the patient with HIV (viral load 6,060 copies/mL, CD4+ T-cell count 570 cells/mm^3^) and initiated antiretroviral therapy. She had no other underlying conditions or notable medical history and reported no international travel or contact with animals. She denied swimming in any water sources or using river or freestanding water for day-to-day purposes; she also denied interacting with any contacts, either in Soweto or Mthatha, with similar complaints or tuberculosis. 

On examination, we found a nontender, 5 mm, mobile, polypoid mass in the right nostril that appeared to adhere to the anterior, superior aspect of the nasal septum near the mucocutaneous junction. Results from the remainder of her ear, nose, and throat examination, as well as examinations of her eyes and pharynx, were unremarkable. We found no cervical lymphadenopathy. 

**Figure 1:**
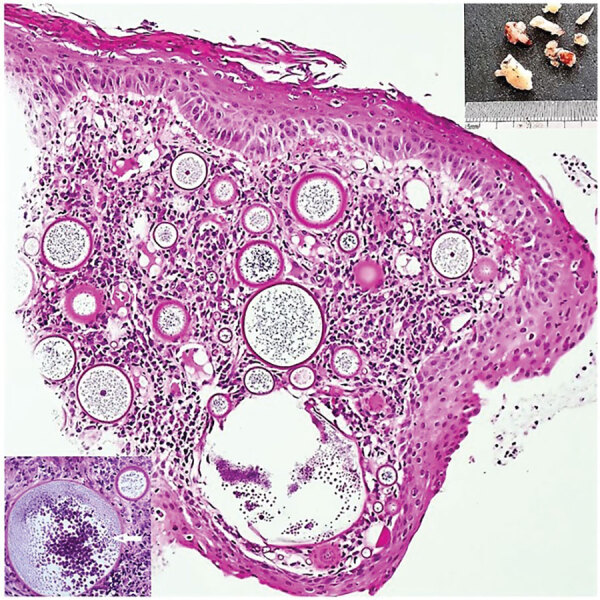
Results of testing in a 24-year-old Black woman with rhinosporidiosis, South Africa. Squamous mucosa with numerous thick-walled sporangia in the subepithelial region amid subacute inflammation. Hematoxylin and eosin stained section; original magnification ×100. Upper right inset shows polypoid solid fragments of tissue; lower left inset depicts sporangia enclosing endospores maturing centripetally (white arrow). Insets: original magnification ×200.

We initiated treatment with oral amoxicillin/clavulanic acid and requested a computed tomography scan of the head and neck to assess the vascularity and amenability for biopsy of the mass. The scan showed a nonenhancing, soft tissue mass in the right nasal vestibule arising from the anterior septum (Appendix Figure 1). A sample of the friable mass from the biopsy sent for histologic examination revealed multiple, variously sized, spherical subepidermal structures, the largest with thickened walls. Contents varied from a single, central acidophilic structure to numerous basophilic spheres that developed centripetally (Figure 1). 

**Figure 2:**
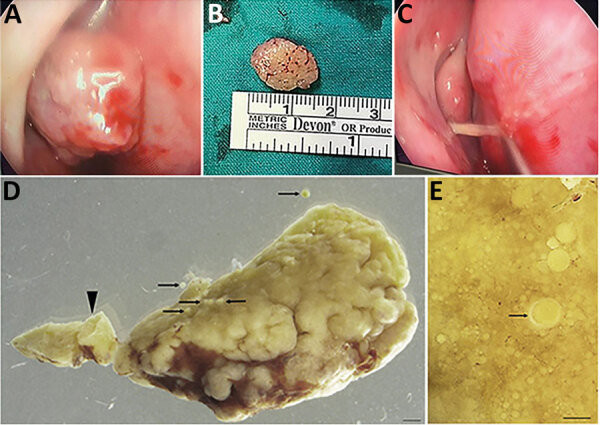
Macromorphology of excised recurrent nasal polyp from a 24-year-old Black woman with rhinosporidiosis, South Africa. A) Intraoperative endoscopic image of mass in right nasal cavity. B) Polypoid, oval mass measuring 15 mm. C) Stalk that attached the mass to the nasal septum. D) Portion of the pedunculated polyp (arrowhead) dotted with developing and mature sporangia (arrows). Scale bar = 1 mm. E) Surface of tissue with multiple sporangia in various stages of maturity, with the chitinous wall thickening during maturation (arrow). Scale bar = 150 μm.

Because of postbiopsy recurrence of the mass and recent onset of pain and epistaxis, we scheduled the patient for definitive surgery to obtain a full-thickness sample. During the operation, we found a 15 mm polypoid mass in the anterior nasal cavity (Figure 2, panels A, B) attached by a stalk to the anterosuperior aspect of the septum (Figure 2, panel C). The stalk did not extend past the mucosa of the nasal septum, so the perichondrium was not macroscopically involved. We fixed the polyp for further microscopic examination (Figure 2, panels D, E; Appendix Figure 2). Examination of the rest of the patient’s nasal cavities were unremarkable. Her postoperative course was uneventful, but she was not available for further follow-up. 

We performed a panfungal PCR test on a section of formalin-fixed paraffin-embedded polyp from the nasal lesion. In the PCR, we amplified fungal DNA for internally transcribed spacer genes (Appendix) and confirmed 98.8% identification and 83% coverage with *Rhinosporidium* spp. (GenBank NCBI accession no. PP060009). Phylogenetically, sequences clustered with *Rhinosporidium* sp. (ex *Canis familiaris*) obtained from a dog (Appendix Figure 3). 

Rhinosporidiosis is an indolent, generally benign, polypoidal infection occurring in humans and other mammals, amphibians, and aquatic birds (*1*,*2*). Host distribution reflects freshwater habitats, the environment most commonly associated with disease acquisition. Rural riverine and agricultural communities have reported the highest incidence (*3*). Other risk factors associated with human infection include contact with stagnant and silted water (typically through swimming or bathing), dust and soil, and contaminated hands or clothes; low socioeconomic status is also considered a risk factor (*3*). Highest incidence is reported in male persons <40 years of age, presumably because of increased exposure as a result of the nature and extent of their outdoor activities (*4*,*5*). The eukaryotic pathogen typically infects exposed nasal, ocular, or genitourinary tract mucosal membranes, with rare reports of cutaneous or disseminated spread in both immunocompetent and immunocompromised patients (*1*,*4*,*6*,*7*). The etiologic agent is *R. seeberi* (class: Mesomycetozoea). Mesomycetozoea comprises a unique group of microbes phylogenetically positioned between fungi and animals, presenting the taxonomic conundrum of a parasite that is neither sporozoan nor fungal, but appears to have features of both types of organism (*8*,*9*).

*Rhinosporidium* grows slowly in host tissues, so infection and clinical manifestations may be temporally distant. Patients with nasal or nasopharyngeal lesions manifest intermittent epistaxis, nasal obstruction, nasal mass, or nasal discharge (*3*,*4*). Clinical differential diagnoses include neoplasms, nasopharyngeal carcinomas, inverted papillomas, primary sinonasal tuberculosis, and nasal angiofibromas (*5*). Diagnosis of rhinosporidiosis is made histologically; sections show multiple sporangia, 50 to >450 μm in diameter, in various stages of maturity. During maturation, chitinous-walled sporangia contain numerous developing endospores 2–10 μm in diameter (*8*,*9*). The nucleated nature of the pathognomic structures precludes identifying the causative agent as *Microcystis*, a gram-negative, phototrophic prokaryote associated particularly with eutrophic lacustrine environments (*8*). 

Treatment of rhinosporidiosis is limited to the surgical removal of polyps and electrocauterization at the attachment base; some clinicians prescribe a prolonged postoperative course of diaminodiphenyl sulfone (dapsone) alone or as part of a multidrug antimicrobial regimen (*4*–*7*). Although not curative, those adjuvants are thought to impede sporangial and endospore maturation. Refractory cases may occur because of incomplete excision, infection of the traumatized surgical sites by released endospores, or reinfection from an endospore reservoir (e.g., lymph) in disseminated cases (*4*,*5*). Recurrence, dissemination to adjacent anatomic sites, and local secondary bacterial infections are the most frequent complications (*3*). Although rhinosporidiosis is rarely fatal, diagnosis and treatment can be lifesaving when nasal infections seed to the tracheobronchial tree (*6*). 

The highest incidence of rhinosporidiosis has been recorded in tropical zones in India and Sri Lanka, followed by South America and Africa, but sporadic autochthonous cases have been reported from tropical and subtropical regions of all continents except Australia and Antarctica (Appendix Table). Three case series and 5 case reports document cases in South Africa (Table); the first reported case was identified by a physician with clinical experience in southern India (*10*). Most cases have been among male children and teenagers, most with conjunctival infections. Reports from several other countries in Africa, including Cameroon, Ivory Coast, Kenya, Malawi, Tanzania, Uganda, Zaire, and Zambia, are most commonly conjunctival infections. Conversely, data from Rwanda and composite global reports indicate ≈70% of infections are nasal or nasopharyngeal infections (*5*,*9*). Misdiagnosis of nasal rhinosporidiosis in some countries in Africa could account for the predominance of reported conjunctival infection in those countries. 

**Table:** Case reports of rhinosporidiosis in South Africa*

Location of exposure or reporting facility†	Age, y/sex (no.)	Infection site	Date reported	Reference
Driefontein, Ladysmith, KwaZulu-Natal	12/M	Nasal	1951	(*10*)
Edendale Hospital, Pietermariztburg, KwaZulu-Natal	10/M	Ocular	1959	(*11*)
Edendale Hospital, Pietermariztburg, KwaZulu-Natal	12/M	Nasal	1977	(*12*)
Edendale Hospital, Pietermariztburg, KwaZulu-Natal	14/M	Nasal	1977	(*12*)
King Edward VIII Hospital, Durban, KwaZulu-Natal	9–15/M (4), F (2)	4 ocular, 2 nasal	1987	(*13*)
Umtata General Hospital, Mthatha, Eastern Cape	<15/M (3), F (3)	6 ocular	2005	(*14*)
Sefako Makgatho Health Sciences University, Ga-Rankuwa, Gauteng	17/M	Nasal	2017	(*15*)
Chris Hani Baragwanath Academic Hospital, Johannesburg, Gauteng	24/F	Nasal	2022	This study

*All patients were Black persons from Africa. †Name of facility at date of publication.

Despite the diagnostic simplicity of rhinosporidiosis, it is unknown if *Rhinosporidium* might have a noninfectious saprophytic developmental phase or natural hosts; how long spores are viable also remains unknown. In addition, the potential role of climate change on the epidemiology of rhinosporidiosis in South Africa is a topic for future research.

In conclusion, our study adds information about the epidemiology and diagnosis of rhinosporidiosis. Because the disease might be misdiagnosed by clinicians who are unaware of its clinical characteristics, providing education could improve rates of accurate diagnosis, leading to better disease surveillance and control efforts. 

Appendix Additional information about case report of nasal rhinosporidiosis in South Africa.
