# Supplementary material for: Case Report of Nasal Rhinosporidiosis in South Africa
Source: Emerg Infect Dis. 2024 Apr;30(4):766–9. doi: 10.3201/eid3004.240018 (PMC10977841; doi:10.3201/eid3004.240018)
Supplement: Appendix — Additional information about case report of nasal rhinosporidiosis in South Africa. [file 24-0018-Techapp-s1.pdf]

Article DOI: https://doi.org/10.3201/eid3004.240018
EID cannot ensure accessibility for Appendix materials supplied by authors. Readers who have difficulty accessing Appendix content should contact the authors for assistance.

Case Report of Nasal Rhinosporidiosis in South Africa

Appendix

Methods

Microscopy

Routine protocols were used for tissue processed for light microscopy. For the scanning electron microscopy (SEM) illustrated below, small pieces of tissue no larger than 4 mm^3 were fixed in 2.5% glutaraldehyde (EM grade) in 0.1 M sodium cacodylate buffer (pH 7.01, osmotically adjusted with the addition of 0.01 M calcium chloride, 0.1 M sucrose and 0.01 magnesium chloride). After four days, the tissue blocks were rinsed three times in fresh buffer, post-fixed for 2 hours in 1% osmium tetroxide in buffer, repeatedly rinsed in fresh buffer, and dehydrated in a graded ethanol series at hourly intervals. Once in absolute ethanol, the tissues were stored until usage of an SEM was available. Some of the dehydrated tissue pieces were cut with a razorblade under liquid nitrogen (a 'primitive' cryo technique), then all tissue was chemically dried with hexamethyldisiloxane. Critical point drying, which is preferable for fungal samples (*1*) was not available. Dried specimens were mounted on stubs with carbon adhesive, sputter coated with platinum, and viewed on a Zeiss Supra 55 VP Field Emission Scanning Electron Microscope at 2 KV.

Molecular

The following primers were used for the *LSU* PCR:

LROR: 5' ACCCGCTGAACTTAAGC 3'

LR5: 5' TCCTGAGGGAAACTTCG 3'

And for the *ITS* PCR:

ITS1: 5' TCCGTAGGTGAACCTGCGG 3'

ITS4: 5’ TCCTCCGCTTATTGATATGC 3’

ITS1: 5’ TCCGTAGGTGAACCTGCGG 3’

ITS2: 5’ GCTGCGTTCTTCATCGATGC 3’

And for the human beta globin PCR (human specimens):

G1: 5’ GAA GAG CCA AGG ACA GGT AC 3’

G2: 5’ CAA CTT CAT CCA CGT TCA CC 3’

The PCR method of White et al. (*2*) was followed. Sequencing was performed by the NICD core facility, and the analyses were done using NCBI (*3*).

References

1. Gibbons J, Geetha-Loganathan P. Processing Embryo, Eggshell, and Fungal Culture for Scanning Electron Microscopy. J Vis Exp. 2019; (150). 10.3791/60018 PubMed https://doi.org/10.3791/60018

2. White TJ, Bruns T, Lee S, Taylor J. Amplification and direct sequencing of fungal ribosomal RNA genes for phylogenetics. In: Innis MA, Gelfand DH, Sninsky JJ, White TJ, editors. PCR protocols: a guide to methods and applications. London: Academic press; 1990. p315–322.

3. Silva V, Pereira CN, Ajello L, Mendoza L. Molecular evidence for multiple host-specific strains in the genus *Rhinosporidium.* J Clin Microbiol. 2005;43:1865–8. 10.1128/JCM.43.4.1865-1868.2005 PubMed https://doi.org/10.1128/JCM.43.4.1865-1868.2005

4. Brygoo ER, Bermond C, Dodin A. First Madagascan case of rhinosporidiosis [in French]. Bull Soc Pathol Exot Filiales. 1959;52:137–40. PubMed

5. Desta TW, Kedir A. Conjunctival rhinosporidiosis in an Ethiopian child: a case report. Int Med Case Rep J. 2022;15:405–8. PubMed https://doi.org/10.2147/IMCRJ.S365729

6. Gigase P, Kestelyn P. Further African cases of rhinosporidiosis. Ann Soc Belg Med Trop. 1993;73:149–52. PubMed

7. Izimukwiye AI, Mbarushimana D, Ndayisaba MC, Bigirimana V, Rugwizangoga B, Laga AC. Cluster of nasal rhinosporidiosis, Eastern Province, Rwanda. Emerg Infect Dis. 2019;25:1727–9. 10.3201/eid2509.190021 PubMed https://doi.org/10.3201/eid2509.190021

8. Udoye EP, Azonobi RI, Opubiri IR. Ocular rhinosporidiosis in a Nigerian child: a case report and literature review from Niger Delta of Nigeria. J Sci Res Rep. 2014;4:599–604. https://doi.org/10.9734/JSRR/2015/14619

9. Ulzen-Appiah K, Akakpo PK, Brookman SB, Imbeah EG. A rare case of conjunctival rhinosporidiosis mimicking a neoplasm in Ghana. Postgrad Med J Ghana. 2022;11:1–4. https://doi.org/10.60014/pmjg.v11i2.290

10. Harissi-Dagher M, Robillard N, Corriveau C, Mabon M, Allaire GS. Histopathologically confirmed ocular rhinosporidiosis in two Canadians. Can J Ophthalmol. 2006;41:226–9. PubMed https://doi.org/10.1139/I06-013

11. Penagos S, Zapata N, Castro JJ, Hidron A, Agudelo CA. Rhinosporidiosis in the Americas: a systematic review of native cases. Am J Trop Med Hyg. 2021;105:171–5. PubMed https://doi.org/10.4269/ajtmh.20-1411

12. Arseculeratne SN, Sumathipala S, Eriyagama NB. Patterns of rhinosporidiosis in Sri Lanka: comparison with international data. Southeast Asian J Trop Med Public Health. 2010;41:175–91. PubMed

13. Ayub-ur-Rehman MMN. Moallam FA. Rhinosporidiosis. J Coll Physicians Surg Pak. 2012;10:671–2.

14. Nguyen VA, Phan ND, Nguyen TL. Two cases of rhinosporidiosis in the Vietnamese [in French]. Acta Med Vietnam. 1959;3:763–8. PubMed

15. Simuangco SA, Bocobo FC, Lacuna L. Review of literature on medical mycology in the Philippines, 1955–1962. Mycopathol Mycol Appl. 1963;20:145–56. PubMed https://doi.org/10.1007/BF02054886

16. Snidvongs ML, Supanakorn S, Supiyaphun P. Severe epistaxis from rhinosporidiosis: a case report. J Med Assoc Thai. 1998;81:555–8. PubMed

17. Thakur SK, Sah SP, Badhu BP. Oculosporidiosis in eastern Nepal: a report of five cases. Southeast Asian J Trop Med Public Health. 2002;33:362–4. PubMed

18. Alonso MJ, Gomez De Tejada R, Diaz P, Ruiz J, Prieto J. Description of a case of rhinosporidiosis in Spain. Mycopathologia. 1987;97:9–16. PubMed https://doi.org/10.1007/BF00437325

19. Vukovic Z, Bobic-Radovanovic A, Latkovic Z, Radovanovic Z. An epidemiological investigation of the first outbreak of rhinosporidiosis in Europe. J Trop Med Hyg. 1995;98:333–7. PubMed

20. Hussein MR, Rashad UM. Rhinosporidiosis in Egypt: a case report and review of literature. Mycopathologia. 2005;159:205–7. PubMed https://doi.org/10.1007/s11046-004-6388-y

21. Firouz-Abadi A, Moghimi M, Azad Y. Rhinosporidiosis in Iran (Persia)—a study of seventy-four cases. Mycopathol Mycol Appl. 1971;44:249–60. PubMed https://doi.org/10.1007/BF02128638

22. Keskin G, Ustündağ E, Kaçarözkara S, Keskin S. An erosive mass involving the nasal cavity and maxillary sinus: rhinosporidiosis. Kulak Burun Bogaz Ihtis Derg. 2004;13:143–7. PubMed

Appendix Table. Countries reporting autochthonous cases of rhinosporidiosis

Region	Country	References
Africa	Burundi, Cameroon, Central African Republic, Chad, Congo – Brazzaville, Democratic Rep of Congo (Zaire), Ethiopia, Ghana, Ivory coast, Kenya, Madagascar, Malawi, Nigeria, Rwanda, South Africa, Sudan, Tanzania, Uganda, Zambia	*4–9*
Central and North America	Canada, Costa Rica, Cuba, Mexico, Panama, USA	*10,11*
South America	Argentina, Bolivia, Brazil, Chile, Colombia, Ecuador, French Guyana, Paraguay, Venezuela	*11*
Asia	India, Indonesia, Nepal, Pakistan, Philippines, Sri Lanka, Thailand, Vietnam	*12–17*
Europe	Serbia, Spain	*18,19*
Middle East	Egypt, Iran, Turkey	*20–22*

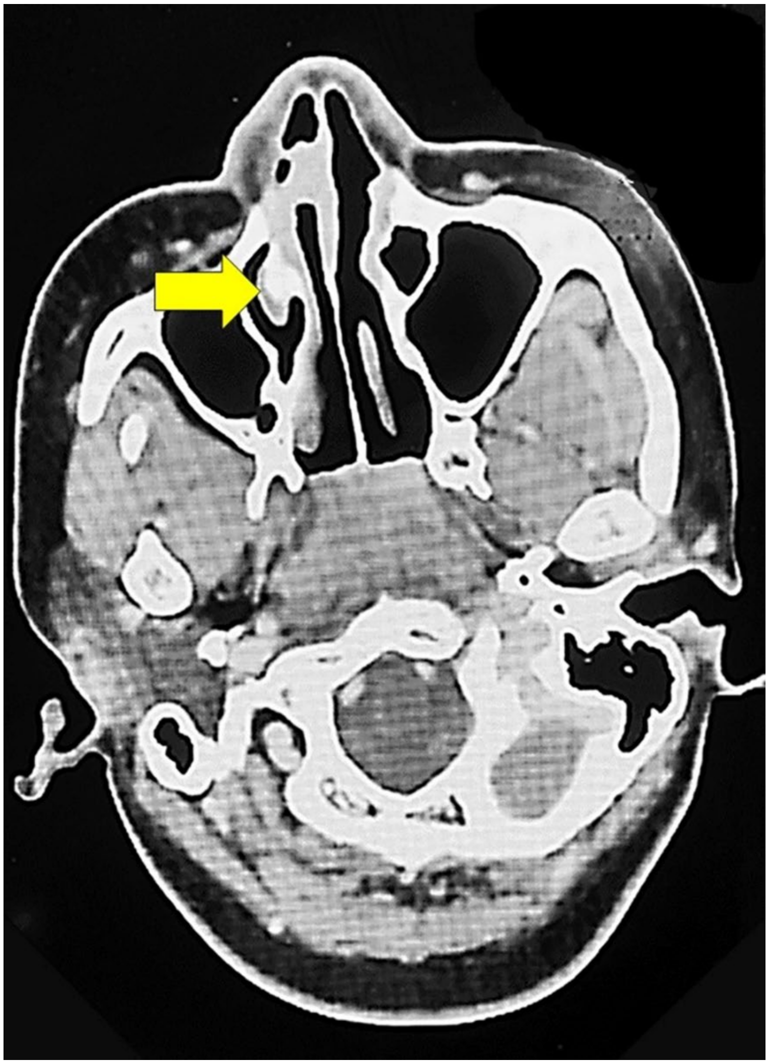

Appendix Figure 1. Computed tomography scan of the nasal cavity, axial section, showing a small homogenous mass with no epithelial extension occupying the anterior part of the right nasal cavity (yellow arrow).

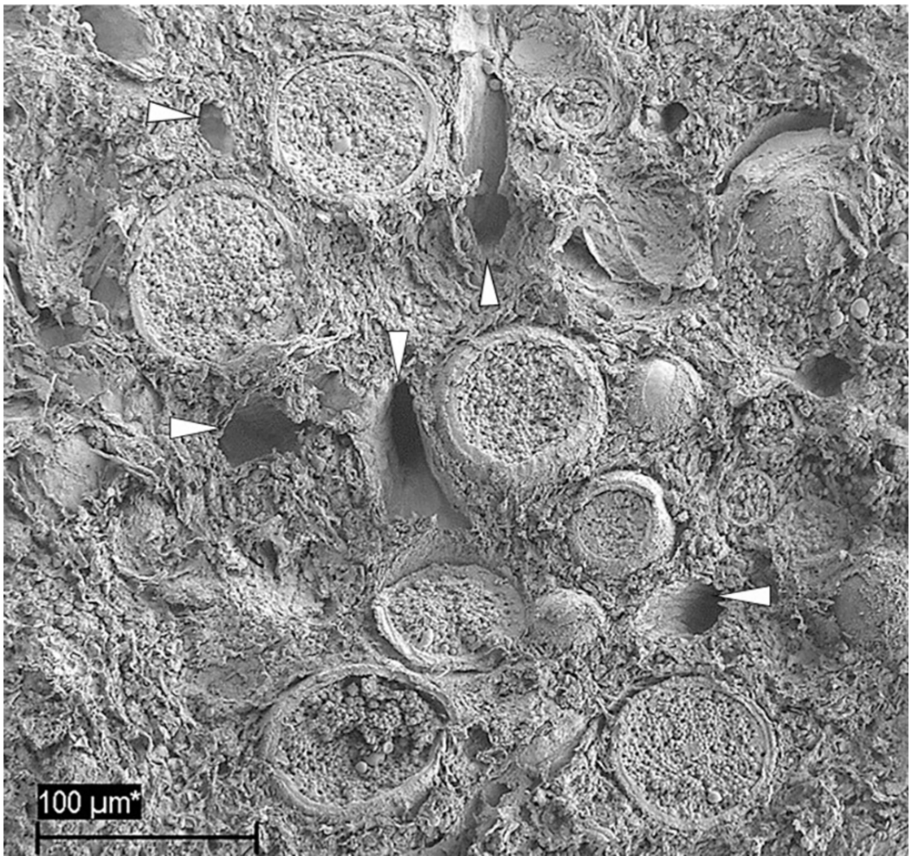

Appendix Figure 2. Scanning electron micrograph of excised nasal polyp (sectioned face) showing the highly vascularized tissue (arrowheads) interspersed with spherical sporangia at various stages of development.

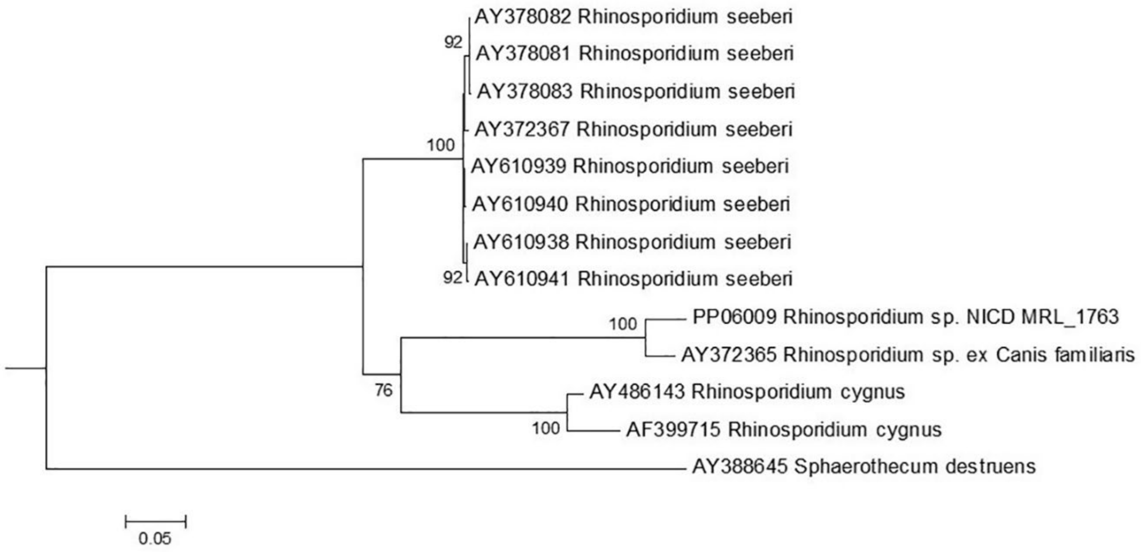

Appendix Figure 3. Phylogenetic relationships of human (*Rhinosporidium seeberi*), dog (*Rhinosporidium sp*. *canis*), and swan (*Rhinosporidium cygnus*) strains based on *ITS* sequences. The phylogenetic tree is constructed using a neighbor-joining algorithm with 1000 bootstrap replications, following the Jukes-Cantor model. The *ITS* sequence from the case patient is denoted as NICD MRL_1763 and clustered with the sequence obtained from a dog (*3*).
